# Incidence and risk factors for liver enzyme elevation during highly active antiretroviral therapy in HIV-HCV co-infected patients: results from the Italian EPOKA-MASTER Cohort

**DOI:** 10.1186/1471-2334-5-58

**Published:** 2005-07-14

**Authors:** Carlo Torti, Giuseppe Lapadula, Salvatore Casari, Massimo Puoti, Mark Nelson, Eugenia Quiros-Roldan, Daniele Bella, Giuseppe Pastore, Nicoletta Ladisa, Lorenzo Minoli, Giovanni Sotgiu, Francesco Mazzotta, Sergio Lo Caputo, Giovanni Di Perri, Gaetano Filice, Carmine Tinelli, Giampiero Carosi, the EPOKA-MASTER Study Group

**Affiliations:** 1 Istituto di Malattie Infettive e Tropicali, Università degli Studi di Brescia, P.le Spedali Civili 1, Brescia, Italy; 2 St. Stephen Centre, Chelsea-Westminster Hospital, 369 Fulham Road, London, UK; 3 I Divisione Malattie Infettive, Spedali Civili di Brescia, P.le Spedali Civili 1, Brescia, Italy; 4 Clinica di Malattie Infettive, Policlinico di Bari, P.za Giulio Cesare 1, Bari, Italy; 5 Istituto di Clinica delle Malattie Infettive, IRCCS S. Matteo, Viale Golgi 2, Pavia, Italy; 6 Divisione di Malattie Infettive, Ospedale SM Annunziata, Via dell'Antella 58, Firenze, Italy; 7 Divisione Clinicizzata di Malattie Infettive e Tropicali, IRCCS S. Matteo, Viale Golgi 2, Pavia, Italy; 8 Istituto di Malattie Infettive, Ospedale Amedeo di Savoia, C.so Svizzera 121, Torino, Italy; 9 Servizio di Biometria ed Epidemiologia Clinica, IRCCS S. Matteo, Viale Golgi 2, Pavia, Italy

## Abstract

**Background:**

The risk of hepatotoxicity associated with different highly active antiretroviral therapy (HAART) regimens (containing multiple-protease inhibitors, single-protease inhibitors or non nucleoside reverse transcriptase inhibitors) in HIV-HCV co-infected patients has not been fully assessed.

**Methods:**

Retrospective analysis of a prospective cohort of 1,038 HIV-HCV co-infected patients who commenced a new HAART in the Italian MASTER database. Patients were stratified into naïve and experienced to antiretroviral therapy before starting the study regimens. Time to grade ≥III hepatotoxicity (as by ACTG classification) was the primary outcome. Secondary outcome was time to grade IV hepatotoxicity.

**Results:**

Incidence of grade ≥III hepatotoxicity was 17.71 per 100 patient-years (p-yr) of follow up in naïve patient group and 8.22 per 100 p-yrs in experienced group (grade IV: 4.13 per 100 p-yrs and 1.08 per 100 p-yrs, respectively). In the latter group, the only independent factors associated with shorter time to the event at proportional hazards regression model were: previous liver transaminase elevations to grade ≥III, higher baseline alanine amino-transferase values, and use of a non nucleoside reverse transcriptase inhibitor based regimen. In the naive group, baseline aspartate transaminase level was associated with the primary outcome.

**Conclusion:**

Use of a single or multiple protease inhibitor based regimen was not associated with risk of hepatotoxicity in either naïve or experienced patient groups to a statistically significant extent. A cautious approach with strict monitoring should be applied in HIV-HCV co-infected experienced patients with previous liver transaminase elevations, higher baseline alanine amino-transferase values and who receive regimens containing non nucleoside reverse transcriptase inhibitors.

## Background

Highly active anti-retroviral therapy (HAART) is associated with a number of serious and potentially life-threatening adverse events, including drug-induced liver injury (so called "hepatotoxicity"). Previous studies demonstrated the association of hepatotoxicity in HIV-infected patients treated with HAART, co-infected with hepatitis C virus (HCV) [1-10]. However, incidence and risk factor data for liver enzyme elevations in large cohorts of HCV-HIV co-infected patients are lacking.

Hepatotoxicity has been associated with any currently used anti-retroviral (ARV) drugs but existing studies fail to demonstrate a consistent association between a particular drug or drug class and the development of subsequent hepatotoxicity, although in a single cohort-study involving HCV positive and negative patients the recent use of nevirapine (within 12 weeks of initiating therapy) and the use of full-dose ritonavir (600 mg bid) have been implicated [9].

It is a general belief that non nucleoside reverse transcriptase inhibitors (NNRTI), especially nevirapine, have a class effect in terms of abnormal liver enzyme levels, but an increased rate of serious clinical (symptomatic) hepatotoxicity has not been comparatively demonstrated in general patient populations yet. Moreover, risk of hepatotoxicity has been shown to be dependent on several concomitant conditions, such as viral co-infection, plasma drug levels, gender and degree of immune damage [11].

Few data are available about the risk of hepatotoxicity during treatment including low-dose ritonavir co-administered with a protease inhibitor (so called "boosted" PI regimens) in comparison with other kinds of regimens. A study, conducted in patients naïve to therapy [12], suggested that HIV-HCV co-infected patients treated with lopinavir/ritonavir-based therapy have similar risk of grade ≥III toxicity when compared with those treated with nelfinavir-based therapy, but the low number of individuals studied and the rigid inclusion criteria adopted preclude generalizations. Another study, conducted in a population of both HCV positive and HCV negative patients [13] compared the incidence of grade ≥III hepatotoxicity in patients receiving their first PI-containing regimen, with or without pharmacokinetic enhancement by low-dose ritonavir, and concluded a similar risk for severe hepatotoxicity between nelfinavir and lopinavir/ritonavir-based regimens, although the number of HCV positive patients was small. Moreover, Meraviglia et al. [14] reported that the risk of hepatotoxicity on lopinavir/ritonavir was moderate and influenced by baseline patient characteristics, including HBV and HCV co-infections.

By contrast, data from a small population of Canadian HIV-positive subjects co-infected with HBV and/or HCV, demonstrated that concurrent use of lopinavir/ritonavir was an independent predictor of grade ≥III alanine amino-transferase (ALT) elevation [15]. Therefore, comparison between different HAART regimens (single-PI, multiple-PI and NNRTI-based) have given inconsistent results in term of liver-tolerability in cohorts in which HIV-HCV co-infected patients are under-represented.

The objective of this paper is to present incidence and risk factor estimates in one of the largest cohorts of HIV-HCV co-infected patients presented so far. As patients were selected for inclusion in the most recent years, it was possible to assess risk associated with modern antiretroviral treatment regimens (containing single-PI, multiple-PI and NNRTI drugs).

## Methods

### Patients

The study consists of an analysis of a prospective cohort of HIV-HCV co-infected patients in the Italian MASTER database. The study was conducted in 5 sites: Brescia, Bari, Pavia, Florence and Turin, in agreement with human experimentation guidelines of the declaration of Helsinki and after approval of the Ethic Committee in each participating centre. Patients were defined as HCV infected by the presence of a positive test for HCV antibodies.

All sequential patients who started any HAART regimen, either PI-or NNRTI-based, from January 2001 to March 2004 have been included in this cohort, provided that a baseline (BL) determination of CD4+ cell count, HIV RNA, aspartate amino-transferase (AST) and ALT levels within 90 days of commencement of therapy were available, and no renal impairment (i.e. creatinine serum level >2 mg/dl), severe liver function test (LFT) elevation (either AST or ALT) or bilirubinaemia ≥5 times the upper limit of normality (ULN) were present. Date of "freezing" of the data-base for the present analysis was September 2004. Patients have been stratified into treatment experienced (E) and naïve (N) for antiretroviral (ARV) drugs before starting the study regimens. Patients were asked to return for clinical and laboratory follow-up (including liver transaminase levels) after 1 month from baseline and at least every 3 months thereafter. The ULN for transaminase was 45 IU/l for ALT levels and 50 IU/l for AST levels in all centres.

### End points

Any increase by ≥5 × ULN in ALT or AST (i.e., grade III hepatotoxicity as by AIDS Clinical Trial Group, ACTG, classification [16]) during the course of HAART was considered to be the primary end-point. Any increase of ALT or AST level ≥10 × ULN (grade IV hepatotoxicity) was considered to be the secondary study end-point [16]. The end-points were not adjusted for BL elevations to increase the sensitivity of capture of hepatotoxicity events. To exclude possible bias, BL LFT elevation was used as a variable in proportional hazards regression models performed.

Finally, a clinical evaluation of patients who experienced grade ≥III or IV hepatotoxicity was performed to rule out that other concomitant causes of LFT elevation were present (i.e., alcohol abuse, defined as >40 g/day; continuing use of recreational drugs; recent lamivudine/tenofovir discontinuation among HBsAg positive patients; use of other hepatotoxic drugs; acute infection with other hepatotropic micro-organisms).

### Statistical analysis

On-treatment (OT) analyses were separately conducted in N and E groups with grade ≥III hepatotoxicity as primary outcome measure. Further analyses were conducted using grade IV hepatotoxicity as a secondary outcome. To ascribe hepatotoxicity to the current study regimens only, follow-up was censored at any modification or stopping of the treatment regimen, or achievement of the study outcomes, whichever came first.

The following factors assessed at BL were tested at proportional hazards regression models for their possible associations with occurrence of the study end-points: gender, age, risk factor for HIV acquisition, nadir CD4+ T-cell count (defined as the lowest value recorded), chronic HBV infection (defined as surface antigen positivity), hepatitis delta virus (HDV) infection (defined as antibody positivity), ALT and AST serum levels, previous LFT elevation to ≥grade III, HIV-RNA, CD4+ T-cell count, previous use of interferon, use and length of exposure to dideoxynucleoside reverse transcriptase inhibitors (DDX) – i.e. stavudine, didanosine and zalcitabine. Type of HAART in the study regimens (single PI, multiple PI and NNRTI-based), and use of DDX, lamivudine (3TC) and tenofovir (TDF) in the current HAART were also considered. Moreover, to account for possible effect of immune reconstitution as risk factor for hepatotoxicity, the CD4+ T-cell count evolution (defined as slope of CD4+ T-cell count since BL throughout the entire follow-up) was imputed in the model as a time-dependent covariate (i.e., a variable that at each follow-up time gives the change in CD4+ T-cell count since baseline).

All variables with a P-value lower than 0.20 at univariate analysis were included in multiple proportional hazards regression model, if clinically meaningful. In all statistical analyses we stated a significance level P ≤ 0.05. Epi Info 3.2 package (Centers for Diseases Control and Prevention, Atlanta 4/2/04) and Egret 2.0.31 package (Cytel Software Corporation, Seattle 1999) were used to perform the analyses.

## Results

### Patients

#### a) Naïve patients

Table 1 shows the main characteristics of the 155 naïve HIV-HCV co-infected patients enrolled in the study. The majority of patients acquired HIV infection through parenteral route (intravenous drug use, IVDU) (67.74%).

**Table 1:** Patient characteristics in naïve and experienced groups

**Charactertistic**	**Antiretroviral naïve (N = 155)**		**Antiretroviral experienced (N = 883)**	
**(quantitative)**	**Mean**	**SD**	**Mean**	**SD**

Age (years)	38.86	6.47	38.91	5.44
Nadir CD4+ (cells/μl)	185.02	115.79	159.99	131.80
CD4+ T-cell count at baseline (cells/μl)	206.75	133.53	357.88	233.05
HIV-RNA at baseline (copies/ml)	177,480	252,619	53,615	168,410
ALT at baseline (x10 IU/l)	6.94	4.14	7.13	4.42
AST at baseline (x10 IU/l)	6.27	3.88	5.87	3.94
CD4+ T-cell slope (cells/μl)	107.86	116.37	44.30	149.48
Exposure to DDX-drugs at baseline (days)	-	-	1,288	1,075

**(qualitative)**	N	%	N	%

Gender (male)	113	72.90	677	76.67
Risk factor for HIV acquisition (former IVDU)	105	67.74	684	77.46
HBsAg positivity	15	10.27	55	6.48
HDVAb positivity	4	2.96	25	2.83
Previous grade ≥III LFT elevation	15	9.68	317	35.90
Previous treatment with IFN	7	4.52	72	8.15
Concurrent use of DDX-drugs	50	32.26	526	59.57
Concurrent use of 3TC	138	89.03	618	69.99
Concurrent use of TDF	17	18.97	136	15.40

Treatment group (Single PI)	32	20.64	202	22.88

NFV	27	84.38	147	72.77
IDV	2	6.25	32	15.84
SQV	3	9.37	19	9.41
Others	-	-	4	1.98

Treatment group (Multiple PI)	50	32.26	322	36.47

LPV/r	42	84	175	54.35
IDV/r	5	10	83	25.78
SQV/r	1	2	48	14.91
Others	2	4	16	4.96

Treatment group (NNRTI)	73	47.10	359	41.62

EFV	42	57.53	165	45.96
NVP	31	42.47	194	54.04

The following abbreviations were used: SD = standard deviation; ALT = alanine amino-transferase, AST = aspartate amino-transferase; DDX = dideoxy-nucleoside analogue drugs (i.e., didanosine, stavudine, zalcitabine); IVDU = intra-venous drug use; IFN = interferon; 3TC = lamivudine, TDF = tenofovir disoproxil fumarate; PI = protease inhibitor, NFV = nelfinavir, IDV = indinavir, SQV = saquinavir, LPV/r = lopinavir/ritonavir, IDV/r = indinavir/ritonavir; SQV/r = saquinavir/ritonavir; NNRTI = non nucleoside reverse transcriptase inhibitor, EFV = efavirenz, NVP = nevirapine.

The following variables were different between drug naive and experienced patients at univariate comparison: nadir CD4+ T-cell count (P: 0.027), CD4+ T-cell count at baseline (P < 0.001), HIV-RNA at baseline (P < 0.001), CD4+ T-cell slope (P: <0.001), risk factor for HIV acquisition (P: 0.009), previous LFT elevation grade ≥III (P < 0.001), concurrent use of DDX-drugs (P < 0.001), concurrent use of 3TC (P < 0.001), use of IDV as single PI (P: 0.002), use of LPV/r (P: 0.04) or SQV/r (P: 0.009) as multiple PI, use of EFV as NNRTI (P: 0.016).

CD4+ T-cell slope was available for 146 patients in naïve group and for 844 patients in experienced group; HBsAg status was available for 146 patients in naive group and for 848 patients in experienced group; HDVAb status was available for 135 patients in naive group and for 833 patients in experienced group.

Among a total of 32 patients who were prescribed a single PI-based regimen, 27 (84.38%) received nelfinavir. All 50 patients who were taking multiple PIs were prescribed ritonavir at low dose (≤200 mg/twice daily), mainly at a dose of 100 mg/twice daily associated with lopinavir (84%). Among 73 patients taking NNRTI-based regimens, 42 (57.53%) received efavirenz.

#### b) Experienced patients

In Table 1, the main characteristics of the 883 experienced HIV-HCV co-infected patients enrolled in the study are illustrated. The majority of patients were male (76.67%), former IVDU (77.46%), and HBsAg negative (93.52%). Two-hundred and two patients (22.88%) were prescribed a single PI; 322 (36.47%) multiple PIs and 359 (41.62%) a NNRTI containing regimen. The single PI most represented was nelfinavir (72.77%), while in the second group almost all patients were prescribed low dose ritonavir (98.75%) plus a full-dose PI, mainly lopinavir (54.35%). In those receiving a NNRTI, 45.96% received efavirenz and 54.04% nevirapine.

### Incidence of relevant hepatotoxicity

#### a) Naïve patients

A total of 20 cases of grade ≥III hepatotoxicity were observed (5 on single-PI, 9 on multiple-PI, and 6 on NNRTI containing regimens), corresponding to an overall incidence of 17.71 per 100 p-yrs of follow-up. Incidence of grade ≥III hepatotoxicity was 20.32 per 100 p-yrs among patients receiving single PI-based regimen, 30.15 per 100 p-yrs among patients receiving multiple PIs and 10.26 per 100 p-yrs in those receiving NNRTI. Additional 90 patients were censored to the follow-up because treatment was changed or stopped for reasons different from hepatotoxicity (19 on single-PI, 27 on multiple-PI, and 44 on NNRTI containing regimens). Forty-five patients were still on the initial HAART regimen at the time of "freezing" the study data base for the analysis. No patient deaths were recorded during the follow-up.

Incidence of grade IV hepatotoxicity was 4.13 per 100 p-yrs (corresponding to a total of 5 events), with 0 per 100 p-yrs in single PI group, 12.49 per 100 p-yrs in multiple PI group and 1.61 per 100 p-yrs in NNRTI group. No significant differences were reported comparing incidences of grade ≥III and grade IV hepatotoxicity in patients receiving nevirapine or efavirenz.

Mean length of observation was 266 days (SD: 263). Mean number of measurements of transaminase levels per year was 5.35 (5.59 for single PI group, 6.16 for multiple PIs group, 4.82 for NNRTI group; t-Student P-value: 0.02).

#### b) Experienced patients

A total of 58 cases of grade ≥III hepatotoxicity were observed (6 on single-PI, 21 on multiple-PI, and 31 on NNRTI containing regimens), corresponding to an overall incidence of grade ≥III hepatotoxicity of 8.22 per 100 p-yrs. Incidence of grade ≥III hepatotoxicity was 3.78 per 100 p-yrs in single PI group, 8.05 per 100 p-yrs in multiple PIs group and 10.82 per 100 p-yrs in NNRTI group. Additional 583 patients were censored to the follow-up because treatment was changed or stopped for reasons different from hepatotoxicity (146 on single-PI, 214 on multiple-PI, and 233 on NNRTI containing regimens). No patient deaths were recorded during the follow-up. Two-hundred and forty-two patients were still on the initial HAART regimen at the time of "freezing" the study data base for the analysis.

Incidence of grade IV hepatotoxicity was 1.08 per 100 p-yrs (corresponding to a total of 8 events), with 0.62 per 100 p-yrs in single PI group, 0.74 per 100 p-yrs in multiple PI group and 1.64 per 100 p-yrs in NNRTI group. As in naïve patients, there was no significant differences in the incidences of grade ≥III or grade IV hepatotoxicity between patients who were prescribed nevirapine and those who received efavirenz.

Mean length of observation was 292 days (SD: 278). Mean number of measurements of transaminase level per year was 4.68 (4.45 for single PI group, 4.81 for multiple PIs group, 4.69 for NNRTI group; t-Student P-value: 0.26).

### Predictors of relevant hepatotoxicity

#### a) Naïve patients

**Figure 1:**
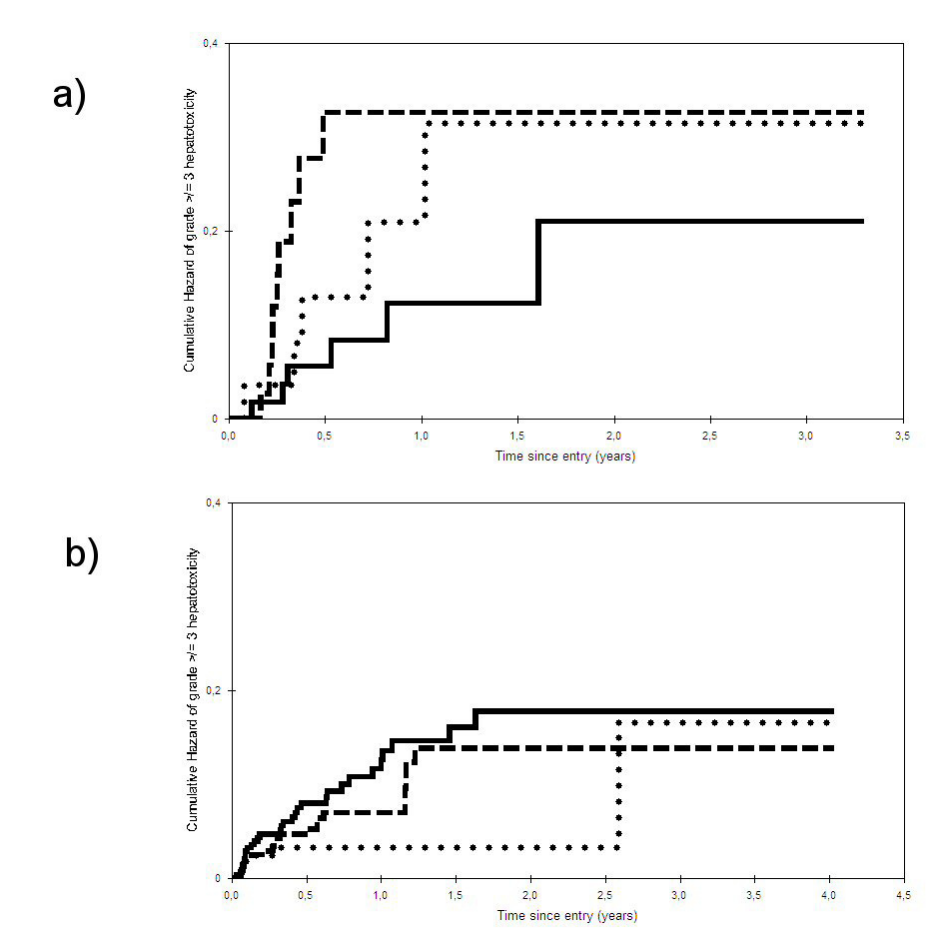
**Time-to-development of grade ≥III hepatotoxicity among naïve (1a) and experienced (1b) patient groups stratified by type of antiretroviral treatment regimens prescribed. **Continuous lines represent patients prescribed HIV-1 non nucleoside reverse transcriptase inhibitors; dotted lines represent patients prescribed single HIV-1 protease inhibitor regimens; dashed lines represent patients prescribed multiple HIV-1 protease inhibitor regimens. P: 0.16 for comparison across antiretroviral naïve patients prescribed different regimens (see panel 1a); P: 0.027 for comparison across antiretroviral experienced patients prescribed different regimens (panel 1b).

Figure 1a represents the risk of developing a grade ≥III hepatotoxicity among naïve patients, stratified by treatment group. At the univariate proportional hazards regression model, time-to-grade ≥III hepatotoxicity resulted significantly associated with BL AST level (HR: 1.12, 95%CI = 1.01–1.23; P: 0.029). No significant correlations were found with use of multiple PI versus a single PI-based regimen (HR: 1.48, 95%CI = 0.49–4.42; P: 0.48) or with use of a NNRTI versus a single PI-based regimen (HR: 0.55, 95%CI = 0.17–1.80; P: 0.32).

When time-to-grade IV hepatotoxicity was considered as dependent variables, none of the factors resulted to be significantly associated with hepatotoxicity. Due to the small number of patients and relatively short follow up, a multivariable hazards regression model was not performed in patients who were antiretroviral therapy naïve, either for the ≥grade III or for the grade IV end-points.

Clinical review did not reveal any concomitant causes for ≥grade III LFT elevations. One patient (an active IVDU), who was assuming a multiple PI-based regimen, had previously developed an acute HBV infection that resolved at the time of hepatotoxicity and another, who was taking a single PI-based regimen, admitted alcohol abuse although AST/ALT ratio did not support an acute alcohol hepatitis.

#### b) Experienced patients

Cumulative risk of time-to-developing a grade ≥III hepatotoxicity among experienced patients, stratified by treatment groups, is represented in Figure 1b. Time-to-grade ≥III hepatotoxicity was significantly associated at the univariate proportional hazards regression model with: male gender (HR: 2.27, 95%CI = 1.03–4.998; P: 0.04), previous use of IFN (HR: 2.29, 95%CI = 1.16–4.53; P: 0.02), previous grade ≥III hepatotoxicity (HR: 4.94, 95%CI = 2.81–8.71; P < 0.001), BL ALT (HR: 1.18 per 10 IU/l higher, 95%CI = 1.12–1.24; P < 0.001), BL AST (HR: 1.11 per 10 IU/l higher, 95%CI = 1.05–1.16; P < 0.001), and NNRTI versus single PI-based regimens (HR: 2.92, 95%CI = 1.22–6.99; P: 0.016).

**Figure 2:**
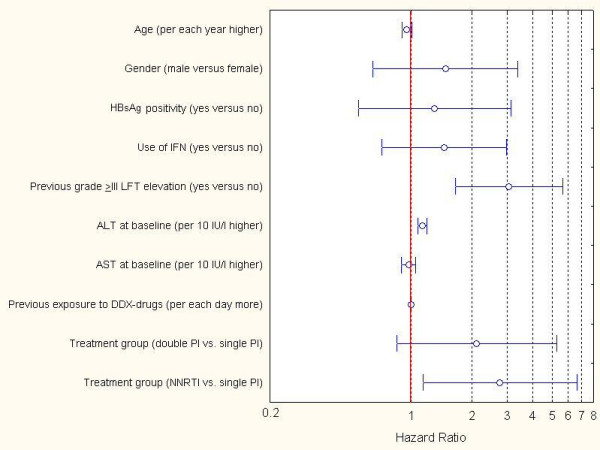
**Results of the multiple proportional hazards regression analysis performed (end-point: grade III LFTs elevation) in experienced patient group. **Circles represent hazard ratio; orizontal lines represent 95% confidence interval. The following abbreviations were used: IFN = interferon; LFT = liver function test; ALT = alanine amino-transferase, AST = aspartate amino-transferase; DDX = dideoxynucleoside reverse transcriptase inhibitors (i.e., didanosine, stavudine, zalcitabine); PI = protease inhibitor; NNRTI = non nucleoside reverse transcriptase inhibitor. Initial models have been run using variables whose data were available for all patients. Results for these variables are reported in the present figure. Further separate models have been performed imputing variables whose data were not available in some patients, however predictive values of variables whose data set was complete did not differ from those obtained through the initial models performed.

Figure 2 shows all variables with a P-value <0.20 in the univariate analyses, which were imputed in the multivariable regression analysis. A history of previous relevant hepatotoxicity (HR: 3.05, 95%CI = 1.65–5.63; P < 0.001), an elevated level of ALT at BL (HR: 1.14 per 10 IU/l higher, 95%CI = 1.08–1.20; P < 0.001) and use of a NNRTI-based treatment (HR: 2.75, 95%CI = 1.15–6.60; P: 0.02) were associated with time-to-grade ≥III LFT elevations.

At the univariate proportional hazards regression analyses performed using occurrence or time-to-grade IV as dependent variables, none of the factors that were tested showed significant associations with outcome.

Among patients who had grade ≥III hepatotoxicity events, those who admitted alcohol abuse were equally distributed in the 3 treatment groups (1 patient for each single PI-, multiple PI-and NNRTI-based regimen, the latter containing efavirenz). No other concomitant causes for grade ≥III hepatotoxicity were found.

## Discussion

This study has a number of strengths but also limitations. A large number of different drug combinations are used in the "real-life" setting but the attribution of an episode of hepatotoxicity to a single ARV agent can be arbitrary. In assigning a patient to a specific treatment arm, physicians may be influenced by many considerations. A subjective evaluation of a high risk of hepatotoxicity may have resulted in prescription of a regimen with an assumed better profile of liver tolerability. Notwithstanding these limitations, observational data reflect the real clinical setting and this is the widest observational cohort of HIV/HCV co-infected patients reported so far. In order to be able to ascribe hepatotoxicity events to ongoing regimen, only a OT approach may be used, discontinuing follow-up when any change to HAART occurs.

Multiple PI-based regimens are not, in this analysis, associated with a significantly higher risk of developing grade ≥III hepatotoxicity when compared to single PI-based regimens, either among patients naïve or experienced to ARV drugs. Ritonavir, whose hepatotoxicity is the clearest defined among PIs, was used rarely at full dosage (1200 mg) in our cohort. When used for pharmacokinetic enhancement in association with another PI, its dosage was in most cases ≤400 mg/day. This low-dose of ritonavir did not appear to influence the liver-toxicity profile of the regimen, thus confirming the report by Sulkowski et al. in a small number of HIV-HCV co-infected patients treated with boosted PI regimens [13].

By contrast, use of NNRTI rather than single PI-based regimens was independently associated, but only among treatment experienced patients, with a higher risk of grade ≥III hepatotoxicity. This finding suggests that careful management of these patients, with a strict follow-up should be performed, especially if other risk factors associated with hepatotoxicity are present.

Among naïve patients, a higher incidence of severe hepatotoxicity and a different pattern of risk factors were reported than those in experienced patients (including the use of NNRTI).

It is possible that more prominent immune-reconstitution occurring in naïve patients than in experienced ones may have increased the overall hepatotoxicity risk, diluting the impact exerted by NNRTI as a cofactor for severe hepatotoxicity. The CD4+ T-cell slope was more pronounced in patients receiving their first line treatment than in experienced subjects. Moreover, the lack of previous drug exposure in naïve patients may have led to prescription of drugs whose potential for hepatotoxicity was less than in the experienced patient cohort, where, due to HIV drug resistance, less choice is available.

It is however important to recognize possible limitations of this study. The low number of patients in the naïve cohort, compared to the experienced cohort, may make the effect of specific drugs in determining hepatotoxicity less evident. Moreover, a different frequency in measuring LFTs, which resulted in more frequent monitoring among naïve patients taking PI-based regimens, may have led to an increased detection of toxicity in that group, although a statistically significant correlation between the frequency of LFT determinations and the risk of hepatotoxicity was not present (data not shown). The low incidence of grade IV hepatotoxicity events did not allow us to perform multivariable analyses. Further studies, with longer follow-up and conducted in a larger series of patients, are therefore necessary in this respect. Lastly, several variables which were not reliably detected in the whole cohort (especially alcohol abuse) should be further evaluated. However, the low prevalence of alcohol abusers in those who had severe hepatotoxicity, which did not cluster in a specific treatment group, may suggest that alcohol abuse was not a major confounder.

## Conclusion

The risk of severe hepatotoxicity was different among experienced and naïve patients, with patterns of risk factors associated with hepatotoxicity being different between these two groups. Amongst experienced patients, elevated ALT level at BL, a history of previous LFT elevation and use of NNRTI-based regimens were independently associated with the development of grade ≥III hepatotoxicity. The choice of multiple PI-rather than a single PI-based regimen did not influence the risk of hepatotoxicity to a significant extent. By contrast, a cautious approach and strict monitoring should be applied in experienced patients with previous hepatotoxicity events, high BL ALT values and prescribed NNRTI-containing regimens, who have the greatest risk of severe liver toxicity.

## Abbreviations

3TC: lamivudine

ALT: Alanine Amino-Transferase

ARV: Anti-Retroviral

AST: Aspartate Amino-Transferase

BL: Baseline

CI: confidence interval

DDX: dideoxynucleoside reverse transcriptase inhibitors

E: Experienced

HAART: Highly Active Anti-Retroviral Therapy

HBsAg: Hepatitis B Surface Antigen

HBV: Hepatitis B Virus

HCV: Hepatitis C Virus

HDV: Hepatitis Delta Virus

HIV: Human Immunodeficiency Virus

HR: Hazard Ratio

IFN: Interferon

IVDU: Intra-Venous Drug User

LFT: Liver Function Test

N: Naïve

NNRTI: Non Nucleoside Reverse Transcriptase Inhibitor

OT: On-Treatment

p-yr: patient-year

PI: Protease Inhibitor

SD: standard deviation

TDF: Tenofovir

ULN: Upper Limit of Normality

## Competing interests

The author(s) declare that they have no competing interests.

## Authors' contributions

CT conceived of the study, participated in its design and coordination and drafted the manuscript. GL participated in the design of the study, in the collection and in the interpretation of data, helped in the statistical analysis and helped in drafting the manuscript. SC performed the statistical analysis and revised the manuscript. MP participated in the design of the study and revised the manuscript. MN helped in the interpretation of data and critically revised the manuscript. EQR revised the manuscript. DB, GP, NL, LM, GS, FM, SLC, GDP and GF participated in the design of the study, in the acquisition of data and revised the manuscript. CT participated in the statistical analysis and revised the manuscript. GC participated in design and coordination of the study, revised the manuscript and collected funding for this study.

## Pre-publication history

The pre-publication history for this paper can be accessed here:


